# Knockdown of Butyrylcholinesterase but Not Inhibition by Chlorpyrifos Alters Early Differentiation Mechanisms in Human Neural Stem Cells

**DOI:** 10.3390/toxics6030052

**Published:** 2018-09-01

**Authors:** Angela K. Tiethof, Jason R. Richardson, Ronald P. Hart

**Affiliations:** 1 Joint Program in Toxicology, Environmental and Occupational Health Science Institute, Rutgers University, Piscataway, NJ 08854, USA; angelatiethof@gmail.com; 2 Environmental Health Sciences, Robert Stempel School of Public Health and Social Work, Florida International University, Miami, FL 33199, USA; 3 Department of Cell Biology & Neuroscience and the Human Genetics Institute of New Jersey, Piscataway, NJ 08854, USA

**Keywords:** neural stem cell, butyrylcholinesterase, chlorpyrifos, notch, HES5

## Abstract

Butyrylcholinesterase (BChE) is the evolutionary counterpart to acetylcholinesterase (AChE). Both are expressed early in nervous system development prior to cholinergic synapse formation. The organophosphate pesticide chlorpyrifos (CPF) primarily exerts toxicity through the inhibition of AChE, which results in excess cholinergic stimulation at the synapse. We hypothesized that the inhibition of AChE and BChE by CPF may impair early neurogenesis in neural stem cells (NSCs). To model neurodevelopment in vitro, we used human NSCs derived from induced pluripotent stem cells (iPSCs) with a focus on the initial differentiation mechanisms. Over the six days of NSC differentiation, the BChE activity and mRNA expression significantly increased, while the AChE activity and expression remained unchanged. The CPF treatment (10 μM) caused 82% and 92% inhibition of AChE and BChE, respectively. The CPF exposure had no effect on the cell viability or the expression of the differentiation markers HES5, DCX, or MAP2. However, the shRNA-knockdown of the BChE expression resulted in the decreased or delayed expression of the transcription factors HES5 and HES3. BChE may have a role in the differentiation of NSCs independent of, or in addition to, its enzymatic activity.

## 1. Introduction

Chlorpyrifos (CPF) is a widely used organophosphorus class pesticide. Because of concerns relating to neurodevelopmental toxicity, the residential and public pest management use of CPF has been eliminated [1]. Epidemiological studies have suggested that CPF exposure is correlated with adverse neurodevelopmental effects involving cognition, behavior, and fetal growth [2,3,4]. Animal studies have evaluated the developmental neurotoxicity (DNT) of CPF using endpoints, including motor activity, cognition, emotion/anxiety, and social interaction [5,6,7,8,9,10]. However, the mechanism(s) responsible for these effects have not been determined, particularly in human neuronal precursor cells, the likely targets in the developing brain.

In animal studies, the daily administration of 5 mg/kg of CPF to pregnant mice between gestation days (GD) 7.5–11.5 resulted in morphological changes, including the thinning of the CA1 and CA3 layers of the somatosensory cortex, the enlargement of the dentate gyrus of the hippocampus, and a decrease in the ratio of neurons and glia in the somatosensory cortex [11]. Similar morphological changes were found in juvenile rats after prenatal CPF exposure [12]. These finding were supported by recent epidemiological data, which compared high and low prenatal exposure groups and found brain changes by magnetic resonance imaging (MRI), including frontal and parietal cortical thinning [13]. Therefore, exposure to CPF during sensitive periods of development may affect the development of the nervous system.

The inhibition of acetylcholinesterase (AChE) has long been considered the primary mechanism of CPF neurotoxicity [14]. However, AChE may have a role in neurogenesis through its enzymatic activity, by interactions with other cellular factors [15,16,17,18], or through an alternate activity as an aryl acylamidase [19].

Both AChE and the pseudocholinesterase, butyrylcholinesterase (BChE), are expressed early in embryonic development [20] and display characteristic spatial and temporal regulation [21]. In neural crest cells, BChE is expressed during mitosis, followed by the increased expression of AChE during migration and differentiation [22]. BChE promotes proliferation prior to differentiation [23,24,25], while AChE may be involved in neurite outgrowth and cell adhesion [17]. Support for the role of AChE in neurite outgrowth is based on the demonstration that blockage of the peripheral site of the enzyme changes the neurite outgrowth density and may influence branching [26]. These non-classical roles for AChE and BChE have been reviewed previously [19,26,27].

Neural progenitor cells (NPCs), also known as neural stem cells (NSCs), are multipotent and can differentiate into neurons, astrocytes, and oligodendrocytes [28]. The neural ectoderm, composed of NPCs, becomes two critical layers, the ventricular zone and the marginal zone, within the fluid-filled vesicle of the developing forebrain. Cell proliferation occurs through vertical cleavage, which expands the NPC population in the ventricular zone and results in the cellular migration and differentiation of one daughter cell. Therefore, in vitro differentiation of NSCs provides an opportunity to assess the potential neurotoxicity of the environmental factors, such as exposure to CPF on aspects of early neurogenesis, including proliferation and differentiation [29,30]. To model the potential effects of CPF on early neural differentiation, we chose to use human NSCs prepared from an induced pluripotent stem cell (iPSC) culture. Our goal was to characterize the NSC system, identify the increased expression of both AChE and BChE, and then use mRNA markers of early commitment to neuronal differentiation to evaluate the effect of the inhibition of cholinesterases by CPF. In addition, we examined whether BChE activity or gene expression was required during early neuronal differentiation.

## 2. Materials and Methods

### 2.1. iPSC and NSC Cultures

Human foreskin fibroblasts were reprogrammed into induced pluripotent stem cells (iPSCs) by retroviral overexpression of transcription factors [31] and were grown feeder-free (i.e., without feeder cells) [32,33]. The iPSCs were characterized and grown as described previously [33].

The NSCs were prepared as described [34], except that Noggin was utilized to inhibit the BMP pathways and promote the differentiation to NSCs [35]. The iPSCS were plated on BD Matrigel™ (BD Biosciences, Billerica, MA, USA) diluted in DMEM/F12 (Gibco, ThermoFisher Scientific, Waltham, MA, USA). The cells were fed every other day with a medium consisting of 50% MTeSR (Stem Cell Technologies, Vancouver, BC, Canada) and 50% neural basal medium (NBM; Neurobasal^®^ medium (Gibco), 2% B27^®^ supplement, 1% insulin–transferrin–selenium (Gibco), 1% N_2_ supplement (Gibco), 2 mM L-Glutamine (Gibco), 0.5% penicillin streptomycin (Gibco), and 500 ng/mL Noggin (Peprotech, Rocky Hill, NJ, USA). Then, 5 days after passage, the medium was changed to 100% NBM with 500 ng/mL Noggin and fed every other day for 10 days. The cells were then passaged onto dishes coated with 20 µg/mL laminin (Sigma, St. Louis, MO, USA) in NBM without Noggin. At approximately 60–70% confluence, the NSCs were switched to a proliferation medium (see below) and grown until about 90% confluent.

The NSCs were cultured in a proliferation medium containing 20 ng/mL recombinant human fibroblast growth factor-basic (FGFb; PeproTech) with 50% DMEM/F12 with GlutaMAX (Gibco), 50% Neurobasal^®^ medium (Gibco), and 0.5X N2 supplement (Gibco) and B27^®^ supplement, minus vitamin A (Gibco). The cells were plated on 1:4 Matrigel™ diluted in DMEM/F12 and passaged with Accutase™ (Stemcell Technologies, Inc., Vancouver, BC, Canada) at approximately 40,000 cells/cm^2^ every 3–4 days.

### 2.2. NSC Differentiation

For the differentiation, the cells were plated in T75 flasks at 13,500 cells/cm^2^ and allowed to grow to confluence. On Day 0, the cultures were switched to a differentiation medium containing 10 ng/mL brain-derived neurotrophic factor (BDNF, PeproTech, Rocky Hill, NJ, USA) in Neurobasal^®^ plus N2 supplement. The cells were harvested using Accutase™ at Day 0 (prior to the initiation of the differentiation), Day 2, Day 4, and Day 6. The cell pellet was resuspended in phosphate buffered saline (PBS) and split 1/3 for RNA and 2/3 for the enzyme assays. The pelleted cells were rinsed well using PBS, and the residual PBS was aspirated prior to freezing at −70 °C.

### 2.3. Chlorpyrifos Exposure

The cells were exposed to CPF (10 µM; *O*,*O*-Diethyl *O*-3,4,6-trichloropyridnyl phosphonothioate; Chem Service Inc., West Chester, PA, USA) starting on Day 0 of the differentiation. The culture medium was changed every other day during the scheduled collection time points. The control cultures contained 1 µL/mL of 100% ethanol.

### 2.4. Lentivirus Production and shRNA Knockdown

For the BChE knockdown, the Mission^®^ shRNA TRC2 lentiviral vector plasmid was purchased from Sigma (TRC number TRCN0000427955, Clone ID NM_000055.2-439s21c1) along with the non-target shRNA control plasmid SHC216. Addgene (Cambridge, MA, USA) third-generation packaging plasmids were transfected at a ratio of 4:2:1:1 of the target vector, MDLg/RRE, VSVg-MD2g, and RSV-REV, respectively, into HEK293T cells. The culture medium was changed daily, harvested for the virus on Day 2 and 3 (at approximately 48 and 72 h post-transfection), and stored at 4 °C. The medium was then centrifuged at 200× *g* to remove cellular debris and virus concentrated by centrifuging at 25,000 rpm for 2 h. The supernatant was removed, and the virus was resuspended in 180 µL DMEM/F12 overnight and stored at −70 °C prior to use.

To quantify the virus, the Sigma-Aldrich Lentiviral Titer p24 ELISA protocol was used, and the viral supernatants were assayed using the Retrotek HIV-1 p24 Antigen ELISA (0801111, Zeptometrix Corp., Buffalo, NY, USA).

For the knockdown experiments, the cultures were seeded at a density of approximately 13,500 cells/cm^2^ in 6-well plate culture dishes and then infected the next day with 6 titer units (TU) per cell in a medium containing 8 µg/mL protamine sulfate (Sigma Aldrich, Saint Louis, MO, USA). The medium was changed the next day to remove the virus and protamine sulfate, and the lysates for RNA were collected on Day 0 and Day 6 of the differentiation.

### 2.5. Quantitative Real-Time Polymerase Chain Reaction

RNA was isolated using the RNeasy^®^ Mini Kit (Qiagen, Hilden, Germany) using on-column DNase treatment. 0.8 µg RNA was reverse transcribed using SuperScript™ (Invitrogen, Carlsbad, CA, USA), using random primers. Quantitative-PCR (qPCR) was performed using Power SYBR^®^ Green PCR Master Mix (Applied Biosystems, Foster City, CA, USA). The relative mRNA expression was calculated using the 2^-ΔΔCt^ method [36], using TBP (TATA binding protein; which had the least variance among all the tested endogenous control targets) as a normalizing gene. The primer sequences are listed in Table 1.

### 2.6. Butyrylcholinesterase and Acetylcholinesterase Enzyme Assays

A 96-well microplate assay was developed from the modification of the methods described previously [5]. The frozen cell pellets were re-suspended in 200–300 µL of 50 mM Tris buffer (pH 7.4 at 37 °C). The cell pellets were sonicated using a Labsonic^®^M (Sartorius Stedim Biotech, Göttingen, Germany). Between 15–30 µg of protein was assayed in duplicate spectrophotometrically, using 1 mM acetylthiocholine iodide (Sigma-Aldrich, St. Louis, MO, USA) as the substrate for AChE activity and 2 mM S-Butyrylthiocholine iodide (Sigma-Aldrich, St. Louis, MO, USA) as the substrate for BChE activity. The chromagen 5,5′-dithio-bis(nitrobenzoic acid) (DTNB, Sigma-Aldrich, St. Louis, MO, USA) was used at a final assay concentration of 0.3 mM, and 10 µM of eserine sulfate (Sigma-Aldrich, St. Louis, MO, USA) was used in duplicate parallel samples to correct for non-enzymatic substrate hydrolysis. The microplate containing the sample, chromagen, and an inhibitor (if required) was pre-incubated for 10 min at 37 °C with shaking. The substrate was added, and the microplate was read at 412 nm for 20 min at 41-sec intervals with mixing. The specific activity was calculated as nmoles product formed per minute per mg protein using the extinction coefficient 14,150 M^−1^ cm^−1^.

### 2.7. Immunocytochemistry and Microscopy

The NSCs were plated on poly-d-lysine (PDL, Sigma)/laminin (Sigma)-coated 12 mm glass coverslips at 400,000 cells/well on Day 2 of the differentiation and allowed to differentiate for 90 days. The cultures were fed with the differentiation media containing 1 µg/mL laminin. On Day 90, the cultures were then fixed for 30 min at room temperature in 4% paraformaldehyde, followed by an incubation for 1 h with 4% normal goat serum and 0.01% triton x-100 in PBS to permeabilize and block. The cells were then incubated at 4 °C overnight with the following antibodies: MAP2 (microtubule-associated protein 2, Millipore, 1:1000), neuronal Class III β-tubulin (TUJ1, Covance, Princeton, NJ, USA, 1:2000), synaptophysin (Millipore, Burlington, MA, USA, 1:500), vesicular glutamate transporter 1 (Vglut, Synaptic Systems, Goettingen, Germany, 1:500), and glial fibrillary acidic protein (GFAP, Dako/Agilent, Santa Clara, CA, USA, 1:1000). Then, the removal of the primary antibody and triplicate PBS washes were performed. The next day, the cultures were incubated for 1 h at room temperature with the following secondary antibodies: Alexa Fluor^®^ 488 conjugate (Goat anti-Rabbit IgG; Goat anti-Mouse IgG2A) and Alexa Fluor^®^ 594 conjugate (Goat anti-Mouse IgG1) at a 1:500 concentration. The nuclei were counterstained with DAPI (0.3 µg/mL, Roche Diagnostics, Indianapolis, IN, USA) and rinsed well with PBS and then distilled water prior to mounting.

### 2.8. Statistical Analysis

The experiments were performed at independent times and cell passages, and each independent differentiation was considered to be a biological replicate. The data were analyzed using a one-way ANOVA model to account for batch variation followed by Tukey multiple comparisons of means to assess changes in mRNA expression and enzyme activity. For CPF- and lentivirus-treated samples, paired *t*-tests were used for each time point to analyze the expression differences between the treatment and control. The data are presented as bar graphs with error bars representing the mean ± standard error of the mean (SEM).

## 3. Results

### 3.1. NSC Model of Early Neuronal Differentiation

**Figure 1:**
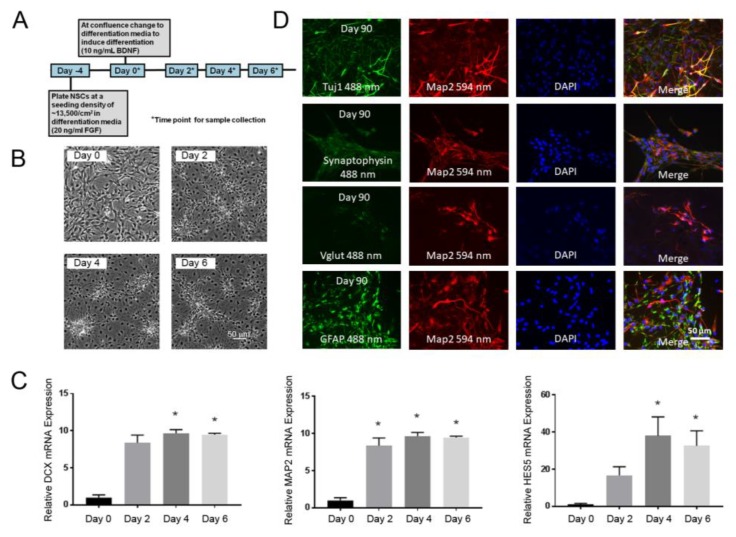
Characterization of the neural stem cell (NSC) model of differentiation. (**A**) The overview of the experimental timeline of the early differentiation. (**B**) Phase-contrast microscopy showing the morphological changes of NSC in the first few days after the induction of the differentiation. (**C**) To assess the early differentiation, the relative mRNA expression of the differentiation markers DCX, MAP2, and HES5 were determined by qPCR. All three markers increased significantly relative to Day 0. The results depict the mean ± SEM (*n* = 3–5) analyzed using a one-way ANOVA model to account for batch variation followed by Tukey multiple comparisons of means (* *p* < 0.05). (**D**) To confirm the multipotency of NSC and that differentiation produces mature neurons and glia, the cultures were fixed and stained immunocytochemically after 90 days of differentiation. All the cultures consisted of cells positive for Tuj1, synaptophysin, and Vglut (punctate staining, counterstained for MAP2) or GFAP (indicative of astrocytes, non-overlapping with MAP2). BDNP: brain-derived neurotrophic factor.

The NSCs were prepared from human iPSCs by switching from a proliferative medium containing FGFb to a medium containing BDNF that favors differentiation (Figure 1A). During the initial six days of the differentiation, the cells transformed from a homogenous appearance to one of clustered cell bodies, with the outgrowth of the processes visible between the clusters (Figure 1B). To assess the NSC differentiation during this initial phase, three mRNAs were chosen as markers (Figure 1C). HES5 was identified from an RNAseq analysis of similarly differentiating human embryonic stem cell (ESC)-derived NSCs [37]. HES5 is a repressive transcription factor, which regulates neuronal differentiation and, along with HES1, is an effector of the Notch pathway [38]. MAP2 is a cytoskeletal protein that is associated with microtubules in neurons [39]. Doublecortin (DCX) is a microtubule-associated protein, which is expressed by migrating neuroblasts during differentiation [40]. All the mRNAs exhibited significant upregulation on Day 4 and Day 6, consistent with neuronal differentiation. The results indicate that the NSC began the process of differentiating into neurons within 2–6 days after the BDNF addition and FGFb withdrawal, and this provides a model for studying key mechanisms during the initial commitment to neurons.

To confirm the appropriate cellular identity of multipotent NSCs, that they produce neurons and glia, we examined the cultures differentiated for much longer periods of time for the production of mature neuronal morphologies and markers. In our previous experience with stem cell-derived NSCs [37], we learned that ~90 days normally produces the robust expression of markers to evaluate the neurons and astrocytes. Oligodendrocyte markers, such as MBP, were not evaluated. Cultures immunocytochemically stained after 90 days of differentiation indicated cells expressing the neuronal markers βIII-tubulin (TuJ1), synaptophysin, and vesicular glutamate transporter (Vglut), each overlapping microtubule-associated protein 2 (MAP2), and the astrocytic marker GFAP, which did not overlap MAP2 (Figure 1D). The detection of diffuse synaptophysin immunoreactivity in the cytoplasm was consistent with neuronal expression prior to synaptogenesis, which would produce a more punctate staining. The large number of Vglut/MAP2 double-positive cells was consistent with the presence of glutamatergic neurons in these cultures. Some cells also stained positive for GFAP, which did not co-localize with MAP2, indicating astrocytes. GFAP- and MAP2-stained cells occurred in similar proportions, indicating a mixture of astrocytes and excitatory neuronal lineages. The expression of neuron and astrocyte markers at later time points indicated that the NSCs were multipotent and that the differentiation protocol was effective.

### 3.2. Butyrylcholinesterase mRNA Expression and Activity Progressively Increases During Differentiation

**Figure 2:**
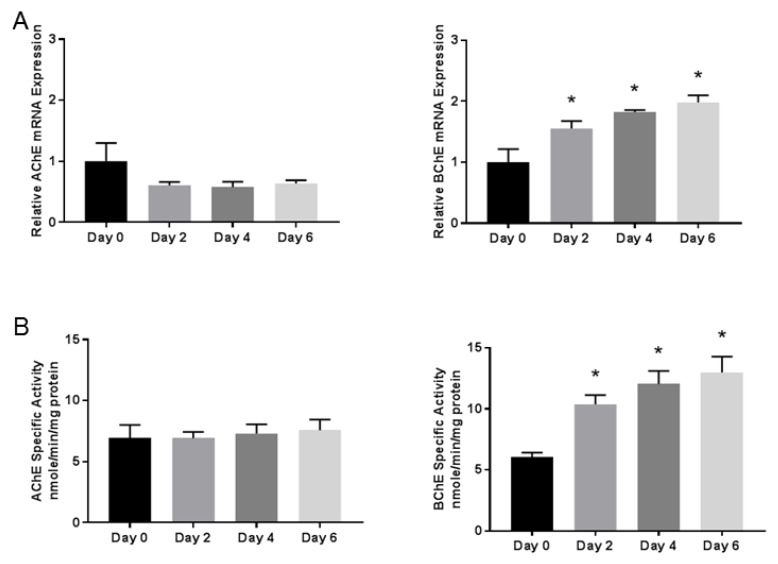
Acetylcholinesterase (AChE) and butyrylcholinesterase (BChE) activity and expression during early NSC differentiation. (**A**) The relative mRNA expression of the enzymes AChE and BChE were determined by quantitative real-time PCR. The BChE mRNA expression increased significantly relative to Day 0 on all subsequent time points shown, while AChE was detected but unchanged over time. (**B**) The AChE and BChE specific activity was assayed using a kinetic spectrophotometric assay. The BChE specific activity increased significantly on all subsequent time points relative to Day 0, mirroring the mRNA expression. The data represent the mean ± SEM (*n* = 5–6). The data were analyzed using a one-way ANOVA model to account for batch variation, followed by Tukey multiple comparisons of means (* *p* < 0.05).

To determine whether AChE and BChE are regulated during early NSC differentiation, mRNA expression and enzyme activities were detected using qPCR and spectrophotometric assays, respectively (Figure 2). During NSC differentiation, the BChE mRNA expression and activity significantly increased during the initial six days. The AChE mRNA expression and activity were unchanged relative to Day 0. This indicated that the BChE mRNA expression and activity were upregulated along with other differentiation markers (HES5, DCX, and MAP2), suggesting that BChE may play a role in the early differentiation of NSCs.

### 3.3. Cholinesterase Inhibition by Chlorpyrifos Does Not Affect Neuronal Marker Expression

**Figure 3:**
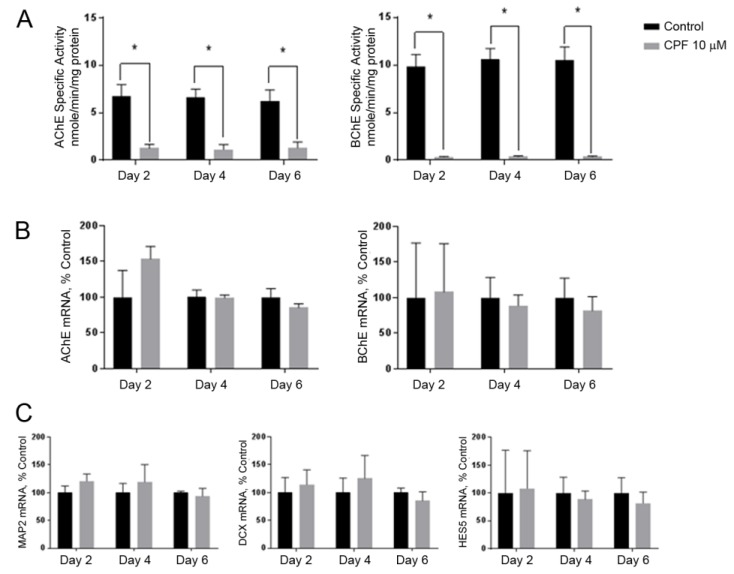
Inhibition of AChE and BChE by chlorpyrifos (CPF). The cell cultures were exposed continuously to 10 µM CPF starting on Day 0 (see Figure 1A **Top right**). (**A**) The AChE and BChE specific activity was assayed using a kinetic spectrophotometric assay. Both AChE and BChE were inhibited by 82% and 92%, respectively. (**B**) The relative mRNA expression levels of the enzymes AChE and BChE and (**C**) the differentiation markers MAP2, DCX, and HES5 were determined by quantitative real-time PCR. There were no significant differences between the control and 10 µM CPF exposure groups. The data represent the mean ± SEM (*n* = 3). The data were analyzed using paired *t*-tests for each time point to account for batch variation. (* *p* < 0.05).

To test the hypothesis that the CPF inhibition of cholinesterase affects early neurogenesis, the NSC cultures were treated with 10 µM CPF. There was no effect on cell viability (as assayed with Alamar Blue^®^, not shown). The CPF inhibited the AChE and BChE activity by 82% and 92%, respectively (Figure 3A). This inhibition of activity did not change the expression of AChE or BChE mRNAs relative to the control group (Figure 3B). However, mRNA markers of neuronal differentiation (HES5, MAP2, and DCX) were not altered by the CPF treatment (Figure 3C).

To assess the potential for the metabolic activation of CPF in NSCs, CYP450 and CP450 reductase (POR) mRNAs were measured by qPCR. Both CYP3A7 and POR mRNAs were detected at all time points, with cycle threshold (Ct) values of approximately 31.5 and 24.5, respectively, indicating a robust signal (results not shown). The likely presence of the metabolizing enzymes and the observed cholinesterase activity inhibition indicates that this model of NSC differentiation is likely to be capable of bioactivating CPF.

### 3.4. Knockdown of BChE mRNA Alters HES5 mRNA Expression

**Figure 4:**
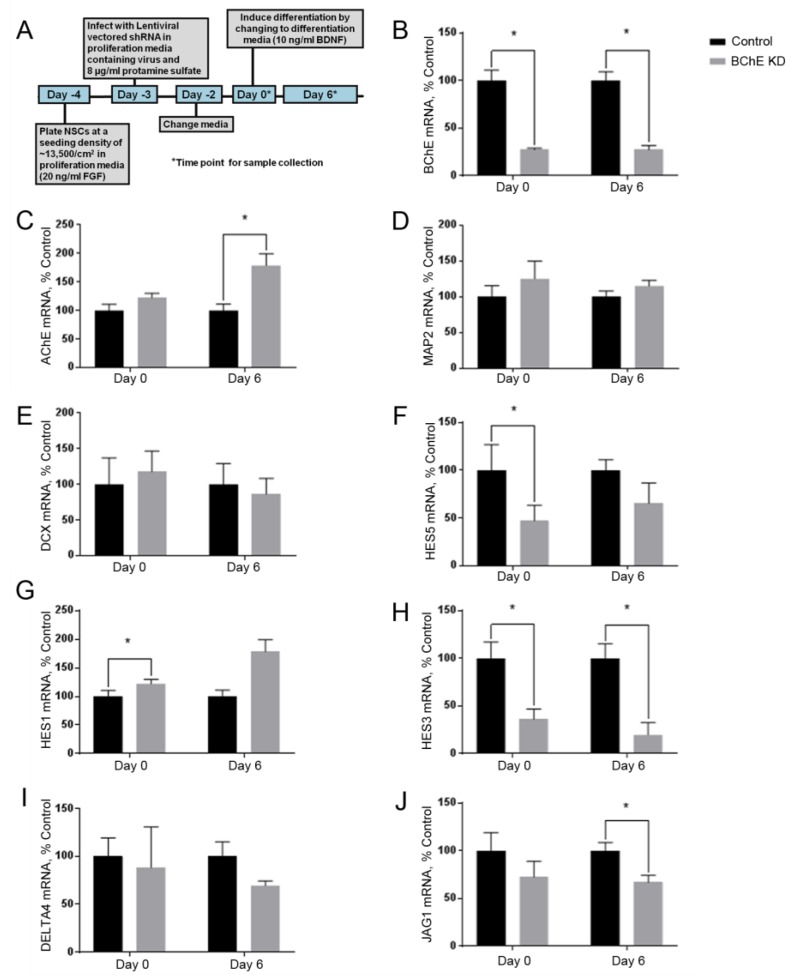
BChE shRNA knockdown. (**A**) An overview of the experimental timeline of the differentiation. The relative mRNA expression levels of the enzymes (**B**) BChE and (**C**) AChE and the differentiation markers (**D**) MAP2, (**E**) DCX, (**F**) HES5, (**G**) HES1, (**H**) HES3, the Notch ligands (**I**) DELTA4, and (**J**) JAG1 were determined by qPCR. The BChE knockdown was calculated to be 72%. The AChE expression was significantly increased on Day 6 relative to the control. Of the early differentiation markers, only HES5 was significantly decreased on Day 0 of the differentiation, but the components of the Notch signaling pathway (HES1, HES3, and JAG1) were affected by the BChE knockdown. The data represent the mean ± SEM (*n* = 4). The data were analyzed using paired *t*-tests for each time point to account for batch variation. (* *p* < 0.05).

Because the CPF inhibition of the BChE activity did not affect the markers of early differentiation, we considered whether the BChE expression was required for normal differentiation. To test this alternative hypothesis that the non-enzymatic functions of BChE regulate neurogenesis, the NSCs were infected with lentivirus expressing BChE or the negative control shRNA 72 h prior to the induction of the differentiation on Day 0 (Figure 4A). The knockdown of the mRNA was calculated to be 72% (Figure 4B). The BChE knockdown resulted in a significant upregulation of the AChE mRNA relative to the control on Day 6 (Figure 4C), with no change in MAP2 (Figure 4D) or DCX (Figure 4E) mRNAs but with a downregulation of HES5 on Day 0 (Figure 4F). We conclude that the BChE mRNA knockdown resulted in the perturbed or delayed expression of the differentiation marker HES5.

Because HES5 is regulated by the Notch pathway, we assayed the mRNA expression of the additional Notch pathway genes HES1, HES3, and the Notch ligands JAG1 and DELTA4 following the BChE knockdown (Figure 4G–J). HES1 was upregulated on Day 0 relative to the control (Figure 4G). The Day 0 sample was taken prior to the BDNF addition and FGFb withdrawal. HES3 was downregulated both on Day 0 and Day 6 (Figure 4H), and the Notch ligand DELTA4 was unaffected (Figure 4I), but JAG1 was down regulated on Day 6 (Figure 4J). The results indicate that the BChE mRNA knockdown, but not the inhibition by CPF, produced changes in the mRNA expression of several developmental and Notch pathway genes, suggesting that BChE likely has a non-enzymatic role in NSC differentiation.

## 4. Discussion

We established an iPSC-derived NSC human cell culture model of early neuronal differentiation as a model for testing neurotoxicity in a human neural progenitor. This system was used to probe the DNT of CPF and to assess the function of BChE early in neurogenesis. The NSC model consists of both a proliferative phase and a differentiation phase, with differentiation characterized by morphological changes and increased expression of the neuronal structural markers MAP2, DCX, and the transcription factor HES5. These three markers were chosen from a comprehensive RNAseq analysis of NSC differentiation in an earlier study [37] as preliminary indicators for the changes occurring during the initial phase of neurogenesis. The differentiation was induced by the withdrawal of pro-mitotic FGFb and the addition of differentiation-promoting BDNF. Much later in the process, after 90 days, the cells stained positive for both neuronal markers, including TuJ1, synaptophysin, Vglut, and MAP2, as well as the non-overlapping astrocytic marker GFAP (Figure 1C), as direct proof that the NSC cultures were multipotent. This model also displayed the expression of both CPF targets BChE and AChE, although only the BChE mRNA expression and activity was increased during the six days of differentiation. Of course, the cholinesterase activity in these isolated cells may not represent the levels seen during intact brain development, but the increase in BChE likely reflects a component or at least an indicator of a developmental mechanism. BChE is known to be expressed at higher levels than AChE in early embryonic development, and, in neural crest cells, BChE is expressed during mitosis and AChE is expressed during migration and through differentiation [21]. This characteristic early expression of BChE during early differentiation is consistent with the hypothesis that BChE may have a role in early NSC differentiation.

To determine whether the catalytic activity of AChE and BChE is essential for early NSC differentiation, the cells were treated continuously with a concentration of CPF sufficient to inhibit the AChE and BChE activities but not enough to cause overt cytotoxicity. Interestingly, we were able to detect the expression of both the CYP3A7 and CYP450 reductase (POR) mRNAs in the NSC model, likely indicating the capacity to convert CPF to its more toxic oxon. In preliminary studies, we had tested the oxon form of CPF and saw no effect, likely due to interactions of the oxon with the Matrigel layer supporting the NSC cultures. In any case, CPF appropriately inhibited the cholinesterase enzyme activities (Figure 3). No activities other than cholinesterase were investigated, so there remains the possibility that CPF or its oxon may affect another pathway, although no effects on the endpoints chosen for study related to neurogenesis were observed.

Others have devised strategies to test neurotoxicity in cellular models of neural progenitors or neurons. In a study using NSCs derived from human umbilical cord blood to model DNT, minimal toxicity was observed at 10 µM CPF, although the cells differentiated more towards an astrocytic phenotype and showed a partial decrease in viability [41]. Another recent study used a commercial, immortalized human cell line to test the epigenetic effects of CPF in both proliferating and differentiating NSCs, and the toxic effects were not detected at concentrations below 57 µM [42]. Neither study quantified the cholinesterase activity. Another study used adipose-derived stem cells to model CPF toxicity [43] but used a much higher dose of CPF (500 μM), finding decreased viability in differentiating cells. One study examined differentiation outcomes in embryonic stem cell-derived NPCs, finding reduced proportions of glial cells upon exposure to 10–30 μM CPF [44]. Others used neuron-like cell lines, including N2a and PC12 cells, identifying the cellular effects, such as neurite retraction [45,46], which would reflect events much later than the initial NSC differentiation modeled here. Our goal was to identify the specific mechanisms affected early in NSC differentiation, because these events are likely to cause changes in the differentiation choice, such as the reduced glial differentiation [43] or changes in the neuronal circuitry due to shifted differentiation patterns.

The CPF inhibition of both AChE and BChE activities, however, did not alter the expression of the early differentiation markers MAP2, DCX, or HES5 (Figure 3C). The dose of 10 μM CPF was chosen due to the lack of observed cytotoxicity and to result in greater than 80% inhibition of ChEs. However, it is within the range of doses found to alter the eventual balance of mature cell types produced later in development [44]. Our data clearly demonstrate that CPF did not affect the initial molecular mechanisms during early neurogenesis from progenitor cells, even with greater than 80% ChE inhibition observed.

One possibility was that neither AChE nor BChE are required for early neurogenesis. Because we observed an increase in mRNA and activity for BChE during this phase (Figure 2), we considered that the BChE protein may be required for the differentiation of NSCs. To test this, we used lentiviral-encoded shRNA to knockdown BChE. The BChE knockdown reduced the expression of HES5 mRNA by over 50% but did not affect MAP2 or DCX mRNAs, suggesting a selective effect. Because HES5 is known to be regulated by the Notch signaling pathway, we also determined that HES1 and JAG1 were altered (Figure 4G,J). Interestingly, prior to the initiation of the differentiation, the BChE knockdown reduced HES3 (Figure 4H) and HES5 (Figure 4F) mRNA levels and increased HES1 (Figure 4G) mRNA expression, suggesting that endogenous BChE expression affects HES signaling in NSCs. The repressor-type basic helix–loop–helix (bHLH) genes HES1, HES3, and HES5 played a critical role in NSC differentiation and maintenance [47]. Activating the bHLH target genes, including Mash1, Math and Neurogenin, promoted neurogenesis, whereas the repressor bHLH genes promoted gliogenesis, which were formed later in neural stem cell differentiation.

HES1 and HES5 are regulated by the Notch pathway [48], but HES3 is not [49]. HES1 and HES5 can compensate for each other, although HES1/HES5 double knockout mice have shown decreased maintenance of glial cells and increased early neurogenesis [47]. Triple knockout HES1/HES3/HES5 mice have a more severe phenotype, which results in the premature differentiation of stem cells in neurons and a loss of other cell types, including astrocytes and oligodendrocytes [50]. Because HES5 expression was decreased with the BChE knockdown, mediation by Notch pathway proteins was likely. The notch ligand JAG1 was decreased on Day 6 of the differentiation. JAG1 is involved postnatal and adult neurogenesis in the dentate gyrus [51], and astrocytes have been shown to decrease neurogenesis though Notch/JAG1 signaling [52].

Based on these results, we propose that BChE may interact with other proteins in NSCs and early during the initial NSC differentiation through a non-enzymatic mechanism. It has been suggested that ChE may act as cell adhesion molecules (CAMs). CAMs that function in nervous system development have an extracellular domain, which displays sequence homology to ChEs [53], and the X-ray crystallography structure of human BChE had a structural homology with neuroligins [54]. The knockdowns indicated that the BChE antisense treatment reduced proliferation and increased differentiation and apoptosis in chick retinae [23,25] and in a rat oligodendroglia cell line [24]. BChE antisense transfection reduced proliferation, increased protein kinase C (PKC), and pushed the cells towards an astrocytic phenotype. Finally, the knockdown of BChE in R28 cells, a retinal rat cell line with pluripotent characteristics, increased the AChE expression, and this “counter-regulation” was tightly controlled through transcription factors c-fos and P90RSK1 [55]. The BChE knockdown perturbed PKC and ERK signaling, suggesting that coordinated ChE expression is involved in cell fate determination. Further research is required to fully elucidate the relative roles of AChE and BChE protein in NSC differentiation and whether this process may be targeted by environmental toxicants to disrupt NSC differentiation.

In conclusion, we established a human iPSC-derived NSC model of early differentiation to probe the DNT of CPF. Although CPF inhibited the enzymatic activity of both AChE and BChE, no effects on NSC toxicity or on the mRNA expression of early differentiation markers HSE5, MAP2, and DCX were found. On the other hand, the shRNA knockdown of BChE resulted in the decreased mRNA expression of HES5 and changes in the expression of the related genes HES3, HES1, and JAG1. These results suggest a novel and potentially non-enzymatic role of BChE in NSC differentiation mechanisms through the regulation of HES genes, possibly through the Notch pathway.

## Figures and Tables

**Table 1:** List of primers for qPCR. All sequences are from 5′ to 3′.

Gene	Forward Primer	Reverse Primer
ACHE	GGGGCTCAGCAGTACGTTAG	CGGTGGCGCTGAGCAATTT
BCHE	CTTTGTTGCAGAGAATCGGAAATC	CGGTGGCGCTGAGCAATTT
DCX	GACTCAGCAAACGGAACCTCC	GAATCACCAAGCGAGTCCGA
MAP2	TGCGCTGATTCTTCAGCTTG	TGTGTCGTGTTCTCAAAGGGT
HES5	CGGCACCAGCCCAACTCCAA	GCGACGAAGGCTTTGCTGTGC
HES1	GAAAGATAGCTCGCGGCATT	TACTTCCCCAGCACACTTGG
HES3	CTGATGGAGAAAAAGCGCCG	TTCCGGATCTGGTGCGAGTA
DELTA4	TGTGCAAGAAGCGCAATGAC	AAGACAGATAGGCTGTTGGCA
JAG1	GTCTCAACGGGGGAACTTGT	GCGTGCTCAGCAATTTCACA
TBP	AAGACCATTGCACTTCGTGCCC	TGGACTGTTCTTCACTCTTGGCTCC
CYP3A7	TTGAAACACGTCTTTGGGGC	TGAGAGAACGAATGGATCTAATGGA
POR	TGCCAGCGTTTCATGATCAAC	GAGACCCACGATGAGCGAAA
